# Supplementary material for: Australian Aboriginal techniques for memorization: Translation into a medical and allied health education setting
Source: PLoS One. 2021 May 18;16(5):e0251710. doi: 10.1371/journal.pone.0251710 (PMC8130951; doi:10.1371/journal.pone.0251710)
Supplement: S1 File — (PDF) [file pone.0251710.s001.pdf]

Qualtrics Survey Questions

Question	Survey mode
**Memorisation is likely to be an important part of my medical education**	**LS: 1 being strongly disagree to 5 being strongly agree**
**I find memorisation:**	**LS: 1 being very difficult to 5 very easy**
**Specific memory training as a component of medical training would be worth my while**	**LS: 1 strongly disagree to 5 strongly agree**
**I found the memorisation test:**	**LS: 1 being very difficult to 5 being very easy**
**In terms of my performance on the recall test, I think the memorisation technique made my performance:**	**LS: 1 being much worse to 5 being much better**
**In describing the memorisation technique, what 5 words would best describe your experience?**	**1.<br>2.<br>3.<br>4.<br>5.**
**What does *memory* mean to you and why might it be useful to a medical student?**	**Text box (no word limits)**
**Is there anything further you wish to add that might be helpful in this research?**	**Text box (no word limits)**
