# Supplementary material for: Australian Aboriginal techniques for memorization: Translation into a medical and allied health education setting
Source: PLoS One. 2021 May 18;16(5):e0251710. doi: 10.1371/journal.pone.0251710 (PMC8130951; doi:10.1371/journal.pone.0251710)
Supplement: Attachment — Submitted filename: Manuscript Number PONE review 29th Sept 2020.docx [file pone.0251710.s002.docx]

**Manuscript Number PONE-D-20-28231
"Australian Aboriginal techniques for memorisation: Translation into a medical and allied health education setting"**

- Very interesting study and easy to follow and understand
- Good to see memory recall occurred six weeks later and limitations associated with the findings as the Aboriginal memory group had a lower level of recall. It would have been good to see more comments around this finding as the students from this group reported using the technique for other study. I would say the novelty of the approach provided a new way to consider memorising vast amounts of information which was reported in the appendix.
- I would have expected the study to capture ethnicity data of the participants as students with an Aboriginal background may be familiar with the technique. In future studies this may be worth noting even in the follow-up section.
- Good to see the cultural safety component has been adhered to with the inclusion of a co-investigator that was of Aboriginal descent. To increase the cultural safety of the research they could be named in the paper rather than anonymised. Then the process and outcome is increasing the capacity and capability of Aboriginal people within research.

Thank you for the opportunity to review the manuscript. Happy to help again in the future.

Regards

Dr Dianne Wepa

29^th^ September 2020
